# Structure-based model for light-harvesting properties of nucleic acid nanostructures

**DOI:** 10.1093/nar/gkt1269

**Published:** 2013-12-05

**Authors:** Keyao Pan, Etienne Boulais, Lun Yang, Mark Bathe

**Affiliations:** Department of Biological Engineering, Laboratory for Computational Biology & Biophysics, Massachusetts Institute of Technology, Cambridge, MA 02139, USA

## Abstract

Programmed self-assembly of DNA enables the rational design of megadalton-scale macromolecular assemblies with sub-nanometer scale precision. These assemblies can be programmed to serve as structural scaffolds for secondary chromophore molecules with light-harvesting properties. Like in natural systems, the local and global spatial organization of these synthetic scaffolded chromophore systems plays a crucial role in their emergent excitonic and optical properties. Previously, we introduced a computational model to predict the large-scale 3D solution structure and flexibility of nucleic acid nanostructures programmed using the principle of scaffolded DNA origami. Here, we use Förster resonance energy transfer theory to simulate the temporal dynamics of dye excitation and energy transfer accounting both for overall DNA nanostructure architecture as well as atomic-level DNA and dye chemical structure and composition. Results are used to calculate emergent optical properties including effective absorption cross-section, absorption and emission spectra and total power transferred to a biomimetic reaction center in an existing seven-helix double stranded DNA-based antenna. This structure-based computational framework enables the efficient *in silico* evaluation of nucleic acid nanostructures for diverse light-harvesting and photonic applications.

## INTRODUCTION

Programmed self-assembly of nucleic acids offers the unique ability to scaffold chromophore molecules at precise locations and orientations in 3D space to construct DNA-based light-harvesting antennas and wires with custom optical properties (1–12). Proof-of-principle studies have demonstrated long-range energy transfer in single-photonic DNA wires consisting of end-tethered donor–acceptor molecules bridged by intercalating YO chromophores undergoing homo-Förster resonance energy transfer (FRET) (13), FRET in a seven-helix double-stranded DNA (dsDNA) bundle assembled from 19 DNA oligos with 13 scaffolded chromophores to act as a light-harvesting antenna (3) and a megadalton-scale DNA assembly synthesized using the principle of scaffolded DNA origami (14) to control FRET in programmed pathways (15), amongst other examples (4). Incorporation of porphyrin molecules into such nucleic acid-chromophore assemblies provides an attractive route to potentially harvesting photonic energy for chemical synthesis (5,16–18).

Importantly, the 3D spatial organization and conformational flexibility of chromophore molecules functionalized to nucleic acid scaffolds plays a critical role in their exciton transport and light-harvesting properties due to the sensitive nature of FRET interactions to both nanometer-scale inter-chromophore distance and transition dipole moment orientation (19). Coherent energy transfer may additionally emerge when closely packed chromophores interact at close range, requiring quantum mechanical modeling considerations (20,21).

To enable the *in silico* design and evaluation of light-harvesting and photonic nucleic acid nanostructures functionalized with chromophores via chemical conjugation to specific DNA bases (3), DNA base replacement and conjugation (19,22,23) and non-covalent attachment via intercalation or groove binding (24–26), we build on the nucleic acid nanostructure-prediction tool CanDo (27,28) to model the atomic-level position and orientation of nucleic acid base pairs and scaffolded dye molecules and compute their excitonic properties using FRET theory. For validation, we apply the proposed framework to isolated dsDNA molecules that act as either a pairwise FRET cascade (29) or photonic wire (13), a kilodalton-scale seven-helix dsDNA bundle light-harvesting antenna (3) and a megadalton-scale DNA origami nanostructure used as a FRET switch (15). The framework is used to predict photonic properties including exciton transfer efficiency, emission and absorption spectra and effective absorption cross-section. This framework offers the ability to rapidly evaluate the structure-based light-harvesting properties of nucleic acid-based devices *in silico*, to investigate the structure-based origin of anomalies in light-harvesting performance measured experimentally and to perform optimization of light-harvesting properties for functional nucleic acid nanostructures. The model may additionally be used as a basis to explore coherent exciton interactions occurring for densely packed chromophores scaffolded in nucleic acid nanostructures in future work (20).

## MATERIALS AND METHODS

**Figure 1.**
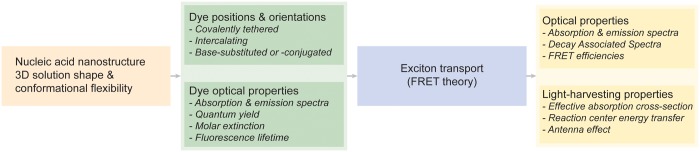
Light-harvesting nucleic acid nanostructure modeling framework. Nucleic acid nanostructure 3D solution shape and conformational flexibility are computed based on input DNA sequence and topology. Scaffolded dye positions and orientations are computed using reference dsDNA and dye atomic structures. Dye positions and orientations are then used together with their individual optical properties to compute time-dependent exciton transfer modeled using FRET theory, as well as emergent optical and light-harvesting properties. This framework enables optimization of functional light-harvesting properties based on iteration of nucleic acid nanostructure 3D solution shape, conformational flexibility, and dye composition and positioning.

Three sequential modeling stages are used to enable the efficient structure-based evaluation of photonic properties of DNA-scaffolded dye assemblies, accounting for both 3D nanostructure solution shape and equilibrium conformational flexibility (Figure 1). The 3D solution shape and thermal fluctuations of the DNA nanostructure are first computed from input nucleic acid sequence and topology using our previously published finite element framework CanDo (27,28). From the finite element model, an atomic-level model consisting of both DNA and scaffolded dye molecules is generated, accounting for sequence-specific nanostructure topology. Finally, FRET-based exciton transport modeling is used to compute the temporal evolution of dye excitation, from which light-harvesting and optical properties are evaluated.

### Nucleic acid nanostructure 3D solution shape, flexibility and atomic-level structure

**Figure 2.**
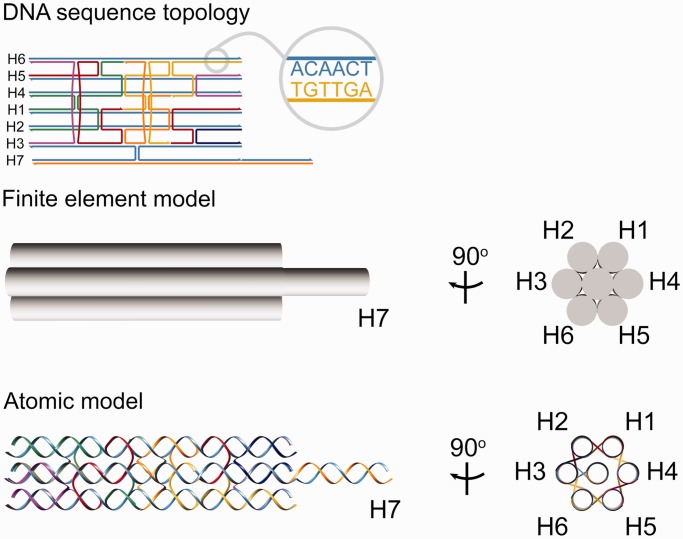
DNA nanostructure atomic model generation and dye modeling. DNA nanostructure sequence topology is used to predict 3D solution structure using the finite element framework CanDo (27,28), which also yields the equilibrium thermally excited conformational ensemble. Base pair-level atomic coordinates are generated from the finite element model using standard reference atomic coordinates. Application to the seven-helix dsDNA bundle antenna from (3) is shown.

The 3D DNA nanostructure solution shape is computed as previously described (27,28) (Figure 2). Briefly, CanDo treats dsDNA as a homogeneous and isotropic elastic rod that is rigidly constrained to its nearest neighbors on a honeycomb or square lattice at specific crossover positions. Each base pair position coincides with a beam finite element node, and neighboring nodes are connected by two-node beam finite elements that model the mechanical stretching, bending and torsional stiffness of dsDNA. A mechanical perturbation followed by non-linear relaxation analysis is used to compute the 3D solution shape using the commercial finite element software ADINA (ADINA R&D Inc., Watertown, MA, USA). Importantly, although the geometric and mechanical properties of dsDNA are typically chosen to be those of B-form DNA—namely, 1100 pN, 230 pN nm^2^ and 460 pN nm^2^ stretching, bending and twisting stiffnesses, respectively—they may equally be chosen to model other forms of dsDNA locally at the single base pair level by changing the corresponding beam finite element geometric and mechanical properties, as described later in the text for dsDNA with intercalating dyes present. Further, the modeling framework presented here is independent of the square and honeycomb lattices assumed in (27,28).

dsDNA atomic coordinates are generated from the finite element model by positioning standard atomic coordinates of the four base pairs A-T, T-A, G-C and C-G at finite element nodes using the topology and sequence information of the DNA nanostructure, the chain identifiers, residue sequence numbers and residue names. The atomic structure of each reference base pair is translated and rotated to the corresponding position and orientation of the pertinent finite element node (Figure 2 and Supplementary Note S1). Subsequent energy minimization and molecular dynamics of the atomic structure that could in principle be performed to refine the atomic model are beyond the scope of the present work.

Equilibrium conformational fluctuations of the DNA nanostructure due to thermal energy are calculated using normal mode analysis (27,28,30) and the Equipartition Theorem (Supplementary Note S2). Although most of the FRET modeling results presented here are for the rigid mechanical ground-state of the nucleic acid nanostructure, the effects of conformational flexibility are examined for the dsDNA-based wire with intercalating dyes. When dsDNA flexibility is accounted for, it is assumed that FRET occurs on timescales that are significantly shorter than the shortest relevant fluctuation timescale of dsDNA, so that FRET may be evaluated for the Boltzmann-weighted DNA conformational ensemble computed from normal mode analysis. Relative dye conformational flexibility with respect to the DNA nanostructure itself that can in principle be incorporated for intercalating and base-substituted dyes, as previously performed by FRET-matrix (19), is ignored in the present work, which assumes that effects of conformational flexibility on dipole–dipole FRET are dominated by the DNA structural scaffold when considered.

### Dye structural model

**Figure 3.**
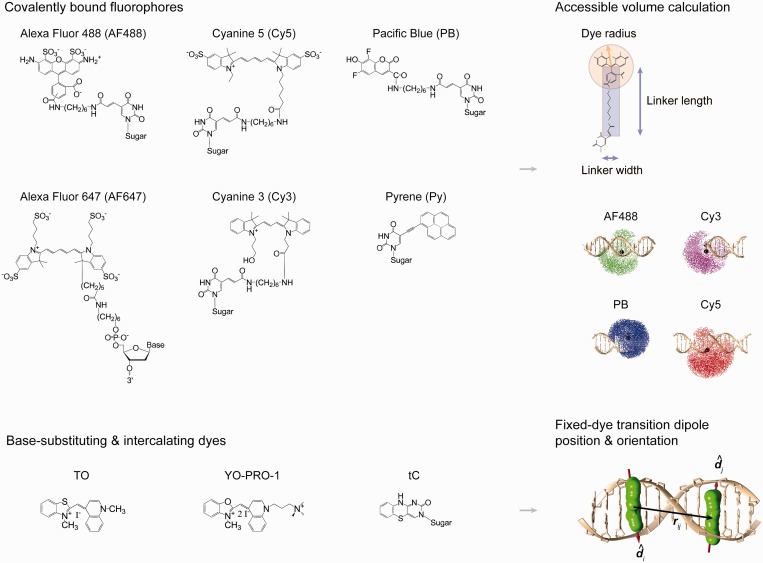
Dye modeling. Dyes are modeled as point transition dipole moments, with prescribed positions and orientations relative to their atomic structure. Covalently bound dyes (top) are modeled using the AV algorithm with geometrical parameters estimated from the dye and linker atomic structure. Geometrical effects of intercalating and base-substituted dyes are accounted for in the finite element model to compute their positions and orientations relative to DNA (bottom).

Dyes are classified into two principal categories based on their scaffolding to dsDNA (Figure 3). In the first, dyes are covalently conjugated to one or more nucleotides via, e.g. C3 or C6 linkers. Examples include cyanine (Cy), pyrene (Py), pacific blue (PB) and Alexa Fluor (AF) dyes. Although these dyes may associate with dsDNA via major or minor groove binding or terminal base pair stacking (31–35), they are assumed not to affect the unstressed geometrical and mechanical properties of B-form DNA so that the structural conformation of the DNA scaffold may be computed in their absence. To compute the distribution of dye positions, the accessible volume (AV) algorithm is used (36,37) (Supplementary Note S3). In this approach, the linker length and dye radius are used to compute the sterically accessible conformational space of the dye, as previously performed for Cy5 and AF488 dyes conjugated to DNA (37). In principle, molecular dynamics simulations or polarized anisotropy measurements could be used to obtain better approximations to the dye orientational and positional distributions relative to DNA (29,37). Importantly, the timescale of the translational diffusion of dyes within the AVs considered in this work is on the order of nanoseconds, setting a lower limit on the timescale of DNA conformational fluctuations that may be accounted for using the FRET model when the Boltzmann distribution of DNA nanostructure conformational states is used.

The second dye category includes dyes that are modeled as fixed in their position and orientation with respect to dsDNA, such as the tC dye family (19), intercalating dyes from the YO and TO families (24–26,38) and intercalated and covalently-conjugated porphyrins (16,39–41). Intercalating dyes affect significantly the geometrical ground-state structure of free dsDNA by elongating and unwinding the canonical B-form dsDNA helix (24,42,43). Effects of intercalating dyes on the geometrical properties (axial rise and twist) of dsDNA are accounted for by modifying the geometric properties of the corresponding beam finite elements. Mechanical properties are assumed to be unaffected but could be modified similarly (Supplementary Note S1). The positions and orientations of intercalating and base-substituted dyes are modeled structurally using 3DNA (44) and their corresponding DNA-dye atomic structures, yielding the transition dipole moment vector center and orientation. Because publicly available experimental atomic-level models of DNA dye structures are currently limited, the TO and YO dyes are both modeled using the TOTO-DNA atomic structure (PDB ID 108D). This assumption is partially justified by the similar geometric properties (axial rise and twist) of YO and TO intercalated in DNA (Supplementary Note S1). Additional PDB structures may be incorporated into the present modeling framework as they become available. The transition dipole moment vector position and orientation of YO-PRO-1 relative to the dye chemical structure is given by (25).

### Exciton transport model

Dyes are modeled as point transition dipole moment vectors with instantaneous position and orientation that are fixed with respect to their configurational ground-state atomic structure (19). Unknown transition dipole moments may be computed from *ab initio* quantum electronic structure calculations (45).

Photoexcitation of a dye molecule creates an electronic excited state called an exciton (46,47). In relatively dense aggregates, this exciton is transferred to neighboring molecules by non-radiative coupling. Assuming relatively weak coupling compared with homogeneous spectral line broadening, which is appropriate for inter-dye separations on the order of or greater than ∼2 nm (48), energy migration may accurately be described using FRET theory (49). In a FRET system of *n* dyes, each dye is energetically coupled to 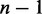 other dyes. The temporal evolution of the exciton population 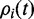 is then described by the master equation,
(1)
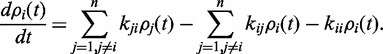



The three terms on the right side of Equation (1) account for the energy transferred to dye 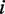 from neighboring dyes 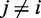, energy transferred from dye 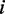 to its neighbors 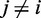 and energy lost due to exciton radiative and non-radiative recombination. 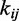 is an off-diagonal element of the rate matrix **K** and represents the asymmetric pairwise FRET rate constant between dyes 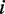 and 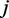 (11),
(2a)
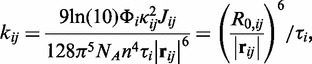

with,
(2b)
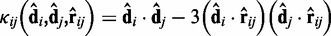

where 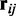 is the position vector directed from the dipole of dye 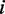 to the dipole of dye 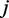, 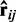 is its corresponding unit vector and 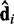 and 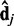 are unit vectors collinear with the corresponding dye transition dipole. 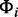 and 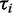 are the quantum yield and fluorescence lifetime of dye *i*, *J_ij_* is the monomeric spectral overlap, *N_A_* is Avogadro’s number, 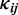 is the dipole orientation factor, *n* is the refractive index of the surrounding medium and *R*_0,_*_ij_* is the Förster critical distance. The diagonal elements of **K**, *k_ii_*, correspond to the total relaxation rate of dye *i*. The structural dependence of the temporal evolution of the exciton population is, therefore, fully described by positions and orientations of the transitional dipole moments, 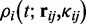.

The master equation [Equation (1)] is solved numerically in MATLAB (The MathWorks, Inc., Natick, MA, USA) subject to the prescribed initial condition on the exciton population, which is assumed to be one for the low-light intensity condition modeled by FRET theory. Thus, the 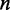 dye system is initially excited assuming a total exciton population equal to one, where the initial 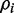 is determined from the molar extinction coefficient of dye 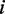 at the irradiation wavelength considered. The temporal evolution of 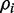 together with the radiation rate, 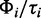, yields the fluorescence intensity. Steady-state properties and transfer efficiency may alternatively be calculated directly in a manner that is equivalent to solving the master equation in steady-state. Fixed excitation rates of the donor dyes are then determined in the same manner as in the calculation of the initial population in the transient case.

The overall FRET efficiency, which is a measure of the effectiveness of exciton transport through the multi-dye system, is calculated from the total integrated fraction of the initial exciton population recombined both radiatively and non-radiatively at the defined acceptor sites,
(3)
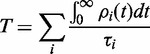

where 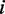 includes only the acceptor sites.

### Dye conformational fluctuations

The conformational fluctuations of the positions and orientations of scaffolded dyes are in general due to (i) the conformational flexibility of the dsDNA scaffold and (ii) the relative conformational flexibility of the dye with respect to the DNA scaffold, which may include relatively small fluctuations if the dye is intercalated, tightly bound or base-substituted/conjugated (19) or significantly larger rotational and translational fluctuations if the dye is conjugated via a flexible linker. In the latter case, the correct FRET averaging procedure depends on the relative timescales of the dye rotational (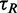) and translational 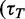) diffusive timescales relative to the FRET timescale (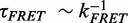) if one assumes that the AV model applies. This is because in the AV model it is assumed that the dye does not transiently bind to dsDNA itself, which may alter both the configurational distribution of positional and orientational states, as well as the dye fluorescence lifetime and quantum yield.

Because FRET efficiency (and any other FRET-related quantity) is highly non-linear with respect to dye position and orientation, it may not be justified to use the mean position and orientation of dyes to compute FRET between dyes with positions and/or orientations that fluctuate significantly in time. In general, when the FRET timescale is much faster than the dye translational and rotational timescales (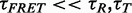), dyes may be considered as configurationally frozen during the energy transfer process, so that the average FRET may be computed by sampling the conformational distributions of dye positions and orientations, 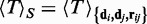, a procedure called static averaging. In the opposite extreme, when 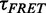 is much slower than the dye translational and rotational motions (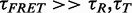), pairwise FRET is averaged from the full distribution of translational and orientational states of the dye that are completely sampled during one pairwise energy transfer event, 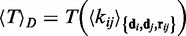. This procedure is called dynamic averaging. In intermediate cases, when only rotation or translation is faster than FRET, such as (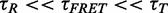), 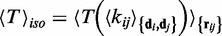, dye positions but not rotations are frozen in performing what is called isotropic averaging (29).

For dyes that are tethered to DNA via long flexible linkers, the characteristic translational diffusion timescale for the dye to sample the entire AV, 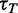, is typically considerably longer than one nanosecond, which is also typically much longer than 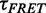. The position of the dye may therefore be assumed to be fixed so that the overall FRET efficiency is given by static averaging over the entire AV (37). On the other hand, 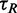 is typically much shorter than 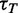, with values 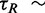
0.1−1 ns (37). For typical dye pairs, this value is smaller than 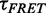, so that dynamic averaging must be assumed in sampling all possible orientations during FRET, resulting in a value of 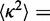
2/3 for isotropic distributions (Supplementary Note S4). Molecular dynamics simulations or polarized anisotropy measurements are needed to more precisely model 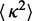 in specific cases, which can deviate significantly from the isotropic value assumed here as a first approximation. Thus, for tethered dyes we use static averaging for dye positions and isotropic dynamic averaging for dye orientations unless otherwise noted. However, when the pairwise distance between interacting dyes is small, 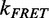 increases strongly, and this approximation may no longer hold. In these cases, static instead of dynamic averaging of the dye orientation must be assumed to ensure accurate calculation of the overall transfer efficiency.

A similar argument for base-substituted and intercalating dyes justifies the use of static averaging for both position and orientation when the conformational dynamics of DNA are considerably slower than 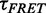. For example, large-scale conformational fluctuations of 12-bp dsDNA occur on 10–100 ns (50). Although these modeling approximations are used to enable the computationally efficient evaluation of FRET properties of high molecular weight multidye DNA-scaffolded systems, dynamical calculations involving either molecular dynamics with explicit solvent for the tethered dyes and the same or coarse-grained Brownian Dynamics for DNA may be performed in future work to more accurately sample the dynamical conformational distributions of states of the DNA dye systems considered (29,51).

### Optical properties and light-harvesting performance

The overall steady-state absorption and emission spectra of the light-harvesting or photonic device may be calculated from the exciton population of each dye and their individual absorption and emission spectra, quantum yield, molar absorptivity and fluorescence lifetime (Supplementary Note S5). Decay-Associated Spectra (DAS) are calculated from the rate matrix 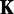 by solving Equation (1) assuming the eigenmode decomposition 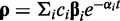, where 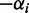 and 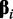 are, respectively, the eigenvalues and eigenvectors of the rate matrix 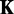 and the amplitudes 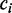 are determined from the initial condition. This enables the calculation of the contribution of each dye to the decay rates (the eigenvalues), and therefore the calculation of the emission spectrum associated with each decay (Supplementary Note S5). In cases where conformational fluctuations are considered, interactions between dyes are computed based on their mean positions.

Two principal parameters that are considered in evaluating the performance of light-harvesting systems are the FRET efficiency (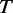) and the wavelength-dependent absorption cross-section 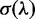. The overall absorption cross-section is characterized by its amplitude and spectral coverage and depends linearly on the number of dyes that absorb at a given wavelength. The effective absorption cross-section at a given wavelength is defined as the product 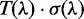, which measures the total exciton population that can be transferred to acceptor dyes at the specific wavelength, 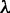. Given a particular incident spectral irradiance 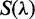, the total power 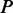 that is transmitted to the acceptor dyes is,
(4)
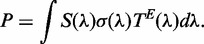



For specific irradiance conditions, 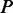 represents the metric that must be optimized. 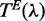 is the energy FRET efficiency, accounting for the fact that the energy of the exciton in the final acceptor dyes is generally different from the exciton initially present in the donor dyes. 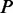 depends on the type of dye, their number (through 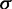), as well as their spatial positioning and orientation through 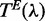. In light-harvesting design applications, optimization of this metric may in principle be performed accounting for both DNA nanostructure shape as well as dye composition and position (Figure 1).

Light-harvesting efficiency is also often characterized experimentally using the antenna effect (3,52). This value represents the ratio of the acceptor fluorescence intensity following excitation of the donor dye(s) relative to the direct excitation of the acceptor dye(s). The antenna effect is, therefore, an empirical measure of the light-harvesting efficiency of the system, and is proportional to the energy transfer efficiency 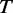 and the ratio of the total molar extinction of the donors and acceptors at the specific wavelengths at which each measurement is taken,
(5)
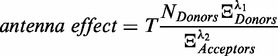

where 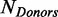 is the number of donor molecules per acceptor, 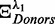 is the molar extinction of the donors at the excitation wavelength 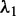 and 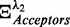 is the molar extinction of the acceptor molecules at the wavelength 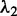. This formula assumes the same photon intensity for both excitations.

## RESULTS AND DISCUSSION

### Application to dsDNA

**Figure 4.**
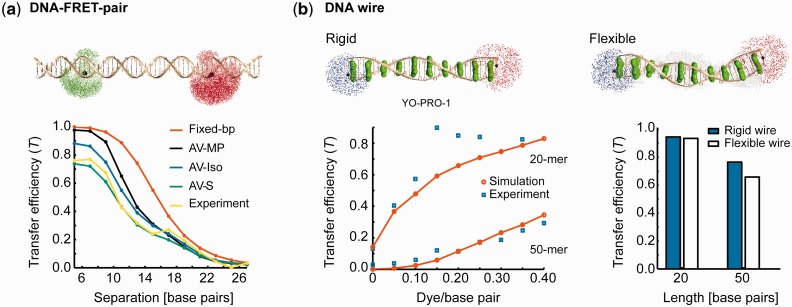
Single dsDNA FRET-pair and photonic wire. (**a**) Pairwise FRET between AF488 and Cy5 tethered dyes as a function of base pair separation. Transfer efficiency is calculated for different types of conformational averaging and compared with experimental results of Wozniak *et al.* (29). Fixed-bp assumes dyes are separated by an integer multiple of the axial rise corresponding to the base pair separation with dynamic rotational averaging. AV-MP assumes FRET to occur between dye mean AV positions with dynamic rotational averaging performed. AV-Iso assumes isotropic averaging, namely static translational averaging and dynamic rotational averaging. AV-S assumes both static translational and rotational averaging during FRET. (**b**) FRET transfer between PB and Cy3 mediated by homo-FRET between intercalated YO-PRO-1 dyes in a dsDNA wire. Transfer efficiency is evaluated for various effective dye/base pair concentrations for the 20-mer and 50-mer wires (left). Simulation results are compared with experimental data from Hannestad *et al.* (13). Both rigid and flexible dsDNA wires are considered theoretically for the fully saturated 50-mer and 20-mer cases (right). dsDNA conformational flexibility is shown using superposition of distinct wire conformations.

Single FRET pairs have been used extensively to probe the structure and conformational dynamics of single-dsDNA molecules (29,36,37). The pairwise FRET efficiency from AF488 to Cy5 covalently conjugated to thymine via C6 linkers exhibits a monotonic decrease beyond ∼10 bp, as expected, given the Förster distance of 5.2 nm and the 0.34 nm/bp axial rise of B-form DNA (Figure 4a) (29). Distinct averaging procedures confirm that static position averaging combined with dynamic rotational averaging best predicts the experimental data assuming rigid dsDNA. In contrast, considering only the mean dye position significantly overestimates the transfer efficiency, *T*. Static rotational averaging could also arguably be used, particularly for lower separations when transfer rates become comparable with rotational diffusion, although all-atom simulations including explicit solvent would be required to refine the dye distribution of states model. Results agree with the model of Wozniak *et al.* (29), who used dye conformational distributions obtained from molecular dynamics simulations rather than the AV method used here. Effects of DNA bending were additionally accounted for by the authors to explain the discrepancy between theoretical and experimental FRET efficiencies in the 16–20 bp separation regime.

Directional photonic energy transport through a dsDNA-based wire was implemented experimentally using homo-FRET interaction between intercalating YO-PRO-1 dyes bridging a single end-terminal donor–acceptor PB-Cy3 pair (Figure 4b) (13). In this system, PB is excited optically and the resulting exciton is transferred to the terminal Cy3 dye through variable numbers of intervening YO-PRO-1 chromophores. For dyes such as YO-PRO-1 that exhibit minimal Stokes shift, efficient homo-FRET is sustained with diffusive inter-dye energy transfer leading to efficient energy transfer up to ∼25 nm (13).

To model FRET in this wire, 1000 dye conformations are simulated for each mean number density of intercalated dyes to account for the variable numbers and positions of the dyes that intercalate randomly along the dsDNA helix (13). Variations of the intercalator quantum yields and fluorescence lifetimes with binding density are also accounted for (Supplementary Note S5). Model results (Figure 4b) are in agreement with both the experimental data and the results predicted previously using a Markov model presented in (13). Interestingly, accounting for flexibility in the DNA double helix due to thermal fluctuations does not significantly affect the transfer efficiency in the 20-mer and 50-mer wires at the maximal packing ratio of 0.5 YO/base pair examined. At this dense packing ratio, energy transfer is generally dominated by nearest-neighbor dyes despite the rotational and transverse fluctuations of DNA (Supplementary Note S6). Although the FRET transfer rate between those pairs varies significantly due to the conformational fluctuations, it remains much higher than the relaxation rate (∼10^8 ^s^−^^1^) and its average value is similar to the rigid wire case, so that the overall transfer efficiency is only weakly affected.

### Application to a seven-helix dsDNA bundle light-harvesting antenna

**Figure 5.**
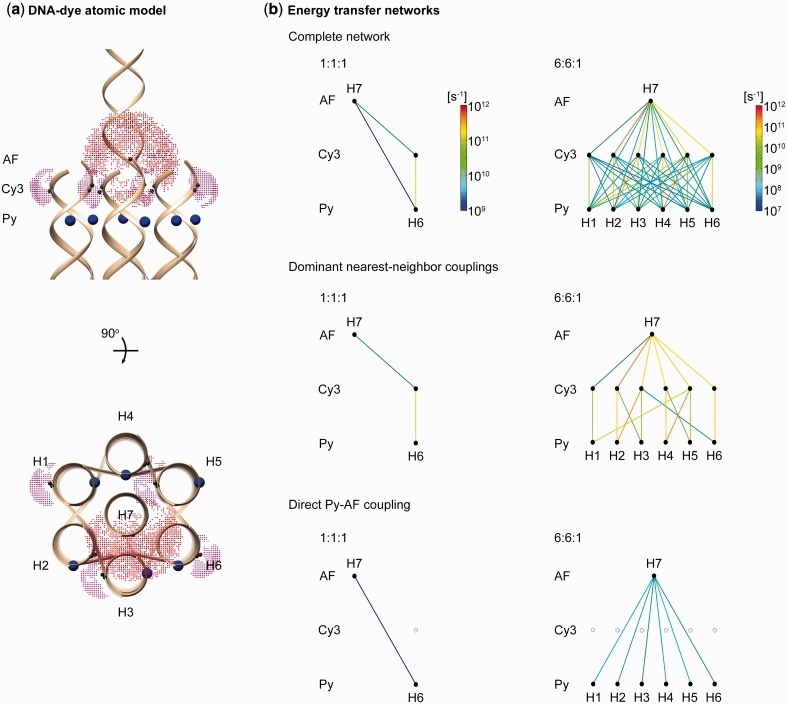
Seven-helix dsDNA bundle antenna atomic model and energy transfer networks. (**a**) Atomic-level structural model of the seven-helix dsDNA bundle light-harvesting antenna with tethered dye AVs shown as point-clouds. Py is linked to DNA with a short, rigid acetylene linker, and is considered to have a fixed position (point-wise AV). (**b**) Energy transfer networks of the 1:1:1 and 6:6:1 antennas.

Dutta *et al.* (3) previously implemented experimentally a light-harvesting antenna consisting of a seven-helix bundle of dsDNA that self-assembles from 19 constituent single-stranded DNA (ssDNA) oligos with variable numbers of chemically conjugated dyes ranging from 3 to 13. In these designs, photon absorption and exciton funneling occurs from a Py donor to an AF647 acceptor via an intermediate, bridging Cy3. In that work, a number of distinct dye organizations that led to different energy absorption and transfer characteristics were examined. In the first, denoted 1:1:1, a single Py chromophore transfers energy to the AF647 acceptor via a single Cy3 bridge (Figure 5a). In a second, 6:6:1 configuration, six Py chromophores transfer energy to a single AF647 via six Cy3 bridging dyes.

In each case, strong coupling between Py and Cy3 dyes is predicted, with major contribution from inter-helix transfer for the 6:6:1 construct, reflecting the fact that the nearest-neighbor distance (1.7 nm for 1:1:1, 1.5–2.4 nm for 6:6:1) is considerably less than the Py-Cy3 Förster distance (3.7 nm) (Figure 5b). Strong transfer from Cy3 to AF is also predicted due to their proximity (3.1 nm for 1:1:1, 1.8–4.3 nm for 6:6:1) relative to the Cy3-AF Förster distance of 5.5 nm. In contrast, considerably lower energy transfer is predicted to occur directly from Py to AF because their separation (3.1 nm for 1:1:1, 2.8–3.7 nm for 6:6:1) is close to the Py-AF Förster distance (3.3 nm). These couplings are calculated from the mean dye positions.

**Figure 6.**
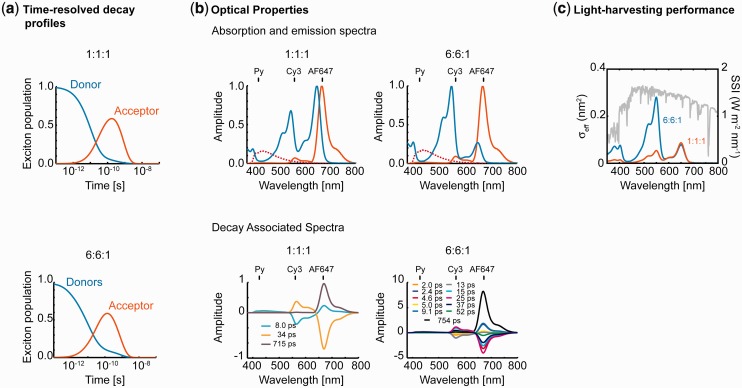
Seven-helix dsDNA bundle antenna optical and light-harvesting properties. (**a**) Time-resolved exciton population decays of the donor (Py) and acceptor (AF) dyes for the 1:1:1 and 6:6:1 antenna designs. (**b**) Steady-state (blue) absorption and (red) emission (top) and DAS spectra (bottom). Isotropic averaging is considered for the steady state spectra, while the dye mean positions are assumed for the DAS calculation. Emission and DAS spectra are calculated assuming initial excitation of Py. Dashed lines indicate Py emission in the absence of Cy3 and AF. The position of the emission peak for each dye is shown in all spectra. Only components with significant amplitudes are shown in the DAS. (**c**) Light-harvesting performance of the 1:1:1 and 6:6:1 antennas. Isotropic averaging is assumed. Effective absorption cross-section and sunlight spectral irradiance is shown (light grey line).

Isotropic averaging is assumed for the calculation of the exciton population and steady-state spectra. Exciton population dynamics for these antenna designs illustrate multiple exponential decays for the 1:1:1 and 6:6:1 constructs (Figure 6a). Steady-state absorption and emission spectra exhibit strong energy transfer from the Py to the terminal AF acceptor along with small residual emission from Cy3, which is consistent with the efficient energy transfer computed earlier in the text (Figure 6b).

DAS spectra also quantify energy transfer from Py to Cy3 and from Cy3 to AF (Figure 6b). Due to the complexity introduced by other types of averaging, DAS is also calculated from the average positions of the fluctuating dyes. Initial energy transfer from Py to Cy3 occurs in ∼8 ps for the 1:1:1 antenna, whereas significantly faster components are present in the case of the 6:6:1 construct (2–13 ps) because each donor can relax via multiple channels. Final transfer to AF occurs in ∼34 ps for the 1:1:1 and 5–52 ps for the 6:6:1 antenna because the Cy3-AF distance is the shortest on the helix H2, which is not present in the 1:1:1 design.

For light-harvesting antennas designed to maximally absorb sunlight, the solar irradiance spectrum may be considered in computing the total power transferred to the reaction center (RC) from the antenna absorption cross-section (Figure 6c). For example, in the seven-helix antenna design only Py is excited at short wavelengths, whereas initial excitation of Py, Cy3 and AF occurs at longer wavelengths, with a probability ratio given by their relative absorption cross-sections at the specific wavelength considered. Finally, the antenna effect quantifies the exciton population transferred to the terminal AF acceptor upon excitation of the donor dyes (Table 1). This result means that 2.72 times more excitons are driven into AF upon excitation of Py than upon direct excitation of AF at their respective absorption wavelengths, respectively, of 380 and 620 nm, for the 6:6:1 antenna. The 6:6:1 antenna is therefore more efficient than the 1:1:1 antenna that has a lower antenna effect of 0.43, which is attributable to its reduced absorption cross-section. Accordingly, the power transferred to the final acceptor under sunlight irradiation is also larger for the 6:6:1 antenna compared with the 1:1:1 antenna. In a functional light-harvesting system that includes a photosynthetic RC in addition to the antenna unit, the total power transferred should match the transformation rate of the RC to yield an efficient design (53).

**Table 1.** Light-harvesting properties of the seven-helix dsDNA bundle antenna

Configuration (Py:Cy3:AF)	Antenna effect	Transferred power under standard sunlight irradiation (fW)
6:6:1	2.72	0.03
1:1:1	0.43	0.01

High transfer rates might reduce the accuracy of isotropic averaging. For example, high calculated transfer efficiency from Py to Cy3 using isotropic averaging is∼96% for the 1:1:1 antenna and range from 96 to 99% for the 6:6:1 antenna, which implies FRET lifetimes in the range of 0.8 ps to 12.5 ns and 0.5 ps to 33 ns, respectively, that can get significantly shorter than typical rotational lifetime 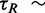
0.1−1 ns (37). Hence, static averaging might be arguably more appropriate for certain pairs of dyes. However, the antenna effects and steady-state spectra calculated using static averaging are not significantly different from those calculated using isotropic averaging (Supplementary Note S7).

Interestingly, the preceding theoretical results suggest a considerably higher Cy3-to-AF energy transfer than that measured experimentally (3), which may be due to a number of sources that are considered in Supplementary Note S8. In future antenna designs, intercalating and base-substituted or -conjugated dyes and porphyrins may be employed instead of tethered dyes due to the ability to more precisely control their positions and orientations, and pairwise single-molecule FRET measurements may be required to resolve differences between theoretical and experimental light-harvesting and optical properties.

### Application to DNA origami

Unlike the preceding examples, natural light-harvesting systems are typically high molecular weight macromolecular assemblies consisting of hundreds or thousands of scaffolded chromophores. For example, the natural photosynthetic unit of purple bacteria forms a spherical vesicle with an approximate diameter of 60 nm and 3000 chromophores (54), whereas the chlorosome of the green sulfur photosynthetic bacterium is an ellipsoidal particle with approximate dimensions of 150 × 50 × 20 nm containing over 10^5^ densely packed chromophores (55,56). In the former case, LH2 subunits act as antennas that transfer excitons on picosecond timescales to LH1 subunits where efficient charge separation occurs in the RC (54,57,58).

**Figure 7.**
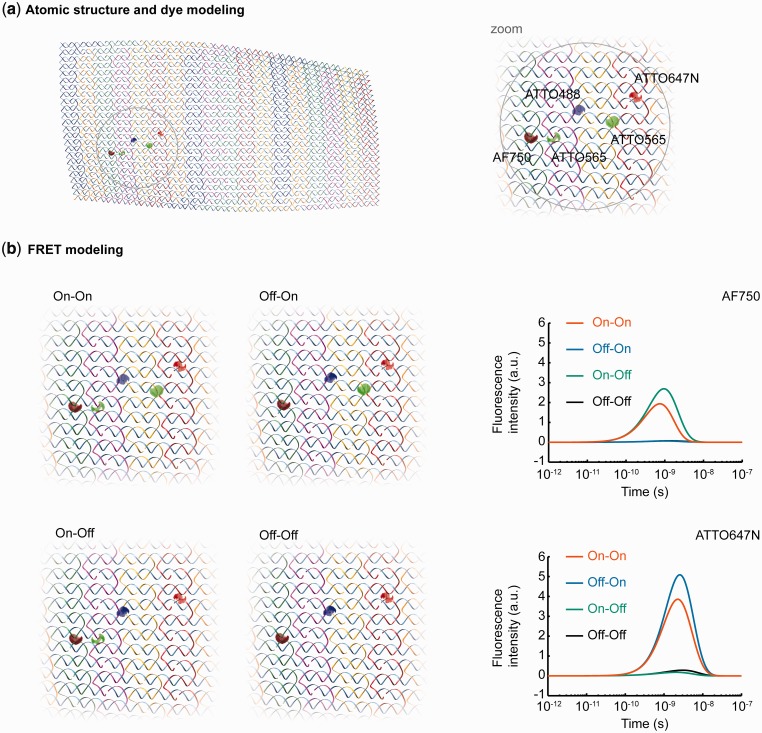
Atomic-structure and exciton transfer in a DNA origami-based FRET switch. (**a**) Top-view of the atomic-level structural model of the FRET switch investigated in (15). AVs of the covalently tethered dyes are shown as colored point-clouds. (**b**) Fluorescence intensity of acceptor dyes AF750 (dark red AV) and ATTO647N (light red AV) after excitation of the donor dye ATTO488 (blue AV) for different configurations of the bridge dye ATTO565 (green AV).

To demonstrate the potential of the proposed structure-based procedure to model and compute FRET transfer for antenna constructs of this molecular weight, we applied our framework to a megadalton DNA origami FRET cascade (15). In this construct, ATTO dyes are used to control FRET in programmed pathways (ATTO488 as donor, ATTO565 as bridge and ATTO647N and AF750 as acceptors). As shown previously, CanDo predicts a non-flat structure of the rectangle in solution due to out-of-balance internal twisting and bending moments that are transferred via crossovers between neighboring helices (Figure 7a). Our sequence-based atomic-structure generation framework renders the detailed base pair-level structure of DNA and conjugated ATTO dyes. While less than one-dozen simple FRET interactions are considered experimentally in this construct (Figure 7b) compared with thousands of dyes that are present in natural light-harvesting systems, the presently proposed FRET modeling framework scales to over 10^4^ dipoles in a straightforward manner with computational complexity that is 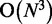 for 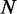 dyes using a direct solver (Supplementary Note S9). Thus, it is feasible using the present computational modeling framework to efficiently explore both DNA nanostructure solution shape as well as multi-dye composition in the future design and optimization of high molecular weight biomimetic DNA-based light-harvesting constructs. However, quantum mechanical modeling will be required for the consideration of coherent inter-dye energy transfer.

## CONCLUDING DISCUSSION

A general computational framework is presented to evaluate exciton transfer amongst dye assemblies that are spatially organized using nucleic acid based scaffolds. Individual dye structural and optical properties are used together with Förster modeling of exciton transfer accounting for three-dimensional DNA nanostructure architecture to compute emergent optical properties. Utility of the framework is illustrated via application to existing DNA-based photonic devices that include a dsDNA wire, a seven-helix dsDNA bundle antenna, and a megadalton-scale DNA origami FRET cascade. Because spatial positioning and orientations of scaffolded dyes play a major role in energy transfer, the proposed modeling framework offers the important ability to design and efficiently evaluate *in silico* distinct DNA-based light harvesting and photonic device architectures prior to experimental synthesis and validation. Further, the atomic-level models that are generated here may be used as input to higher resolution computational modeling approaches including molecular and Brownian dynamics to account for dynamical structural and quantum properties that are not considered in this work. The latter are likely to play an important role in the design of highly efficient light-harvesting antennas such as those found in nature (53,59–61). An important difference between natural light-harvesting systems and those explored in this work is that the former utilize tight inter-subunit protein packing to achieve geometrically precise and stable orientations of chromophore molecules (54). While various strategies including click chemistry as well as covalent and non-covalent binding offer opportunities to achieve tightly packed chromophore assemblies scaffolded purely by DNA (2,62,63), an interesting alternative strategy is to employ programmed hybrid protein-DNA nanostructures for this purpose (64). The atomic models generated by the present framework will be important to enable the rational structure-based design of such hybrid protein-DNA assemblies.

## AVAILABILITY

Atomic models of DNA nanostructures are available as part of the CanDo webserver (http://cando-dna-origami.org). Atomic structure generation, dye AV generation and optical property calculation software is available upon request.

## SUPPLEMENTARY DATA

Supplementary Data are available at NAR Online.

## FUNDING

Office of Naval Research [ONR N000141210621 to K.P. and M.B.]; and the Army Research Office [ARO MURI W911NF1210420 to L.Y., M.B. and E.B.]; are gratefully acknowledged. E.B. additionally acknowledges support from the Natural Sciences and Engineering Research Council of Canada. Funding for open access charge: Office of Naval Research [ONR N000141210621].

*Conflict of interest statement*. None declared.

## Supplementary Material

Supplementary Data
