# Supplementary material for: Structure-based model for light-harvesting properties of nucleic acid nanostructures
Source: Nucleic Acids Res. 2013 Dec 5;42(4):2159–70. doi: 10.1093/nar/gkt1269 (PMC3936709; doi:10.1093/nar/gkt1269)
Supplement: Supplementary Data [file supp_42_4_2159__index.html]

Structure-based model for light-harvesting properties of nucleic acid nanostructures — Structure-based model for light-harvesting properties of nucleic acid nanostructures — Supplementary Data 

# Structure-based model for light-harvesting properties of nucleic acid nanostructures

## Supplementary Data

files

**Files in this Data Supplement:**

- Supplementary Data - zip file
