# Supplementary material for: Structure-based model for light-harvesting properties of nucleic acid nanostructures
Source: Nucleic Acids Res. 2013 Dec 5;42(4):2159–70. doi: 10.1093/nar/gkt1269 (PMC3936709; doi:10.1093/nar/gkt1269)
Supplement: Supplementary Data [file supp_gkt1269_suppl_data.zip › nar-02976-f-2013-File009.pdf]

Structure-based model for light-harvesting properties of nucleic acid nanostructures: Supplementary Information

Keyao Pan†, Etienne Boulais†, Lun Yang, and Mark Bathe
Laboratory for Computational Biology & Biophysics
Department of Biological Engineering
Massachusetts Institute of Technology
Cambridge, MA 02139, USA

SUPPLEMENTARY NOTE 1: Atomic model generation

CanDo calculates the mechanical ground-state 3D solution shape of DNA nanostructures using the finite element method, where each finite element node corresponds to a single DNA basepair and the local finite element beam models the geometric and mechanical properties of dsDNA (1,2). The finite element model therefore provides the Cartesian coordinate, $\mathbf{d}$, and orientation, $\mathbf{R}$, of each basepair, with respect to an arbitrary global reference frame. The basepair chain identifiers and residue sequence numbers for each node are known from the topological design of the DNA nanostructure, represented here using the JSON file format from caDNAno (3). The topological designs of the structures studied in the present work are available in (4-6).

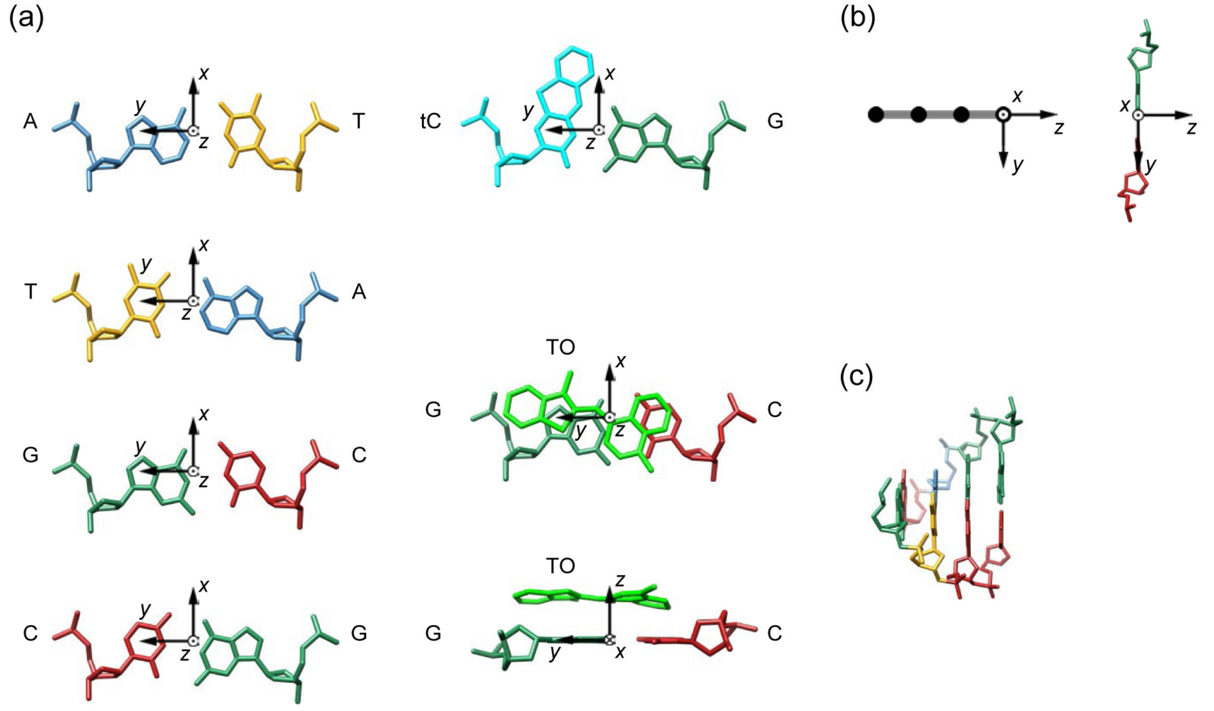

Figure S1. Generation of the atomic model of a DNA nanostructure from the finite element model. (a) Standard reference atomic structures for the four canonical Watson-Crick basepairs, the base-substituted dye tC, and the intercalating dye TO in two views. A basepair frame is defined for each standard reference atomic structure. (b) A simple representative finite element model consisting of four nodes denoted as black dots connected by three beam elements denoted as gray lines. The nodal frame is shown for the right-most finite element node. The atomic model is generated by aligning each basepair frame to the local finite element nodal frame using rigid body translation and rotation. (c) The atomic model corresponding to the finite element model in (b).

Figure S1 illustrates the procedure used to construct the atomic structure of a DNA nanostructure from the finite element model. Standard reference atomic structures for the Watson-Crick basepairs A-T, T-A, G-C, and C-G are generated using Accelrys DS Visualizer Version 3.5 (Accelrys, Inc., Burlington, MA) local basepair reference frame is defined for each standard reference atomic structure using the 3DNA convention (7). For example, the basepair frame for the A-T basepair is the average of two base frames for bases A and T, respectively, which are defined in Table 1 of Olson et al. (8). The basepair frame has its x-axis pointing towards the major groove, its y-axis towards base A, and its z-axis perpendicular to the basepair plane. For each standard reference atomic structure, the centers and axes of the basepair frame align with those of the global reference frame. These standard reference coordinates are subsequently rotated and translated to the orientation and position of the corresponding finite element node using,

$$\mathbf{x} = \mathbf{R}\mathbf{x}_0 + \mathbf{d} \quad [1]$$

where $\mathbf{x}_0$ is the Cartesian coordinate of an atom in the reference basepair aligned with respect to the global reference frame.

The above procedure is also used to construct the atomic model of base-substituted dyes and intercalating dyes in DNA nanostructures. A base-substituted dye is a base analog that replaces one base in the standard basepair atomic structure (9). We assume that replacing a base with a base-substituted dye preserves the mechanical and geometrical properties of the corresponding element in the finite element model. Thus, to incorporate a base-substituted dye, the finite element model does not require modification, and the atomic model is built by applying Eq. [1] to rotate and translate the corresponding reference basepair atomic structure with one base replaced by the dye. We use the tC dye (9) as an example. The tC dye replaces the cytosine in a C-G basepair, and so the standard reference atomic structure is generated by fitting the atomic structure of the tC dye to that of the cytosine in the standard reference atomic structure of the C-G basepair. The standard reference atomic structure of tC-G is then rotated and translated using the finite element model and Eq. [1].

In contrast, intercalating dyes intercalate between two neighboring basepairs, thereby altering the ground-state structure of the dsDNA helix, inducing local elongation and untwisting in the DNA duplex without affecting its bending stiffness in the case of YO-PRO-1 (10). We further assume that its stretching and twisting stiffnesses are not affected either. To model intercalating dyes, the finite element model is modified to account for the local elongation and untwisting caused by the dye. Table S1 provides the measured local elongation and untwisting of two intercalating dyes: YO-PRO-1 and TO (11). For example, intercalation of YO-PRO-1 causes an elongation of 0.34 nm/dye (5) and an untwisting of 24°/dye (10). The corresponding beam finite element with intercalating YO-PRO-1 therefore has a length increase from 0.34 nm to 0.68 nm, a twisting angle decrease from 34.3° to 10.3°, and unchanged mechanical properties. The corresponding atomic structure is built using Eq. [1] to place the standard reference atomic structures according to the finite element output. The atomic structure of the intercalating dye is built by using Eq. [1]. Figure 4(b) in the main text shows the atomic structures of DNA-dye complexes.

Table S1. Local elongation and untwisting caused by intercalating dyes. The values for TO are approximated from Figure S2.

**Intercalating dye**	**Elongation**	**Untwisting**
YO-PRO-1	0.34 nm/dye (5)	24°/dye (10)
TO	0.34 nm/dye (5)	11°/dye (Figure S2)

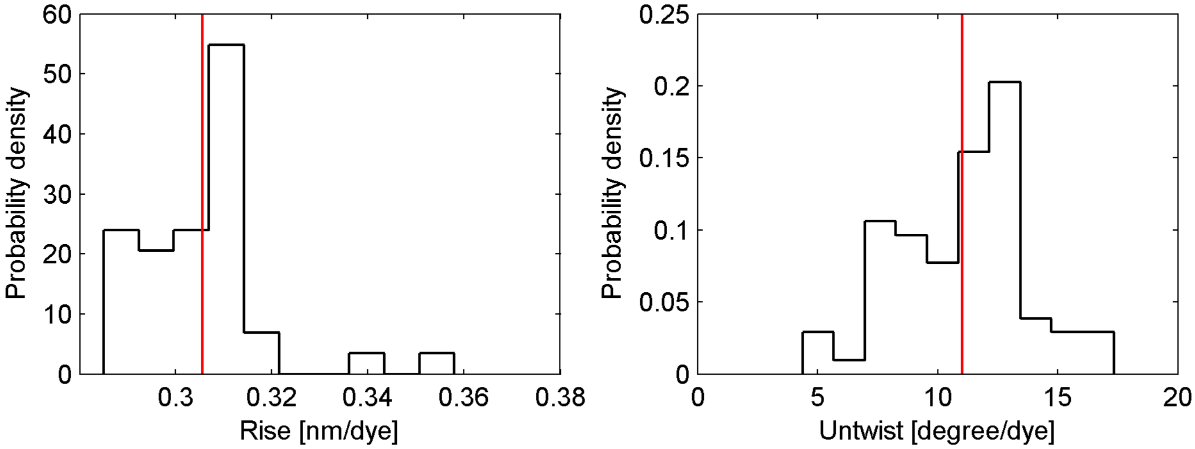

Figure S2. Distributions of elongation and untwisting caused by the intercalating dye TO, calculated from the atomic structure (12) (PDB ID: 108D) using the 3DNA convention (7). The red vertical lines show the mean values of elongation and untwisting.

SUPPLEMENTARY NOTE 2: DNA nanostructure conformational fluctuations

Normal mode analysis (NMA) is used to compute the structural flexibility and corresponding thermal fluctuations of DNA nanostructures at finite temperature, as performed previously (1). To reconstruct a time-varying sequence of conformational states of the DNA nanostructure, which properly samples the Boltzmann-weighted distribution of conformational states but does not represent physically realistic time-scales due to the absence of solvent, the instantaneous thermal fluctuation of each basepair is represented as a time-varying displacement vector $\mathbf{d}^{\mathrm{NMA}}(t)$ and rotation matrix $\mathbf{R}^{\mathrm{NMA}}(t)$ with respect to the global reference frame. Thus, the instantaneous Cartesian coordinates of atom $\boldsymbol{x}$ is given by,

$$\mathbf{x}^{\mathrm{NMA}}(t) = \mathbf{R}^{\mathrm{NMA}}(t)\mathbf{R}\mathbf{x}_0 + \mathbf{d} + \mathbf{d}^{\mathrm{NMA}}(t) \qquad [2]$$

where the rotation matrix $\mathbf{R}$ and the translation vector $\mathbf{d}$ are those from the same equilibrium 3D solution shape as in Eq. [1]. According to Eq. [2], in order to build the atomic structure at time t from the NMA output, the standard reference atomic structures for the A-T, T-A, G-C, and C-G basepairs are first rotated by the rotation matrix $\mathbf{R}^{\mathrm{NMA}}(t)\mathbf{R}$ in the global reference frame and then translated by the vector $\mathbf{d} + \mathbf{d}^{\mathrm{NMA}}(t)$. The basepair chain identifier and residue sequence number for each node is generated with the same topological design, as described in Supplementary Note 1.

For example, consider a DNA nanostructure with m basepairs. Basepair i, $i = 1, \dots, m$, is modeled as a finite element node with three translational degrees of freedom (DOFs) $u_i^{tx}, u_i^{ty}, u_i^{tz}$ along the x, y, z axes and three rotational DOFs $u_i^{rx}, u_i^{ry}, u_i^{rz}$ about the x, y, z axes. The total number of DOFs is $n = 6m$. If $n > 206$, then only the first 206 normal modes with the smallest eigenvalues are calculated, where the eigenvalues and eigenvectors for each normal mode are computed using the commercial software ADINA 8.9 (ADINA R&D, Inc., Watertown, MA).

Here we use a single basepair i to illustrate the modeling procedure. Without loss of generality, we assume that the calculated normal modes are sorted in ascending order of the eigenvalues. The first six normal modes are discarded because they correspond to the rigid-body translation and rotation of the DNA nanostructure. The DNA conformation is homogeneously sampled during a period T_{p}, which equals the period of oscillation in the seventh normal mode. In each time frame, $t = 0.01T_{\mathrm{p}}, 0.02T_{\mathrm{p}}, \dots, T_{\mathrm{p}}$, the initial phase of each normal mode is a random number uniformly distributed between 0 and 2π. The

increments in the six DOFs of basepair i in a time frame are then obtained from the calculated normal modes.

To facilitate downstream analysis, the increments in the six DOFs of basepair i are represented as a rotation matrix $\mathbf{R}_i^{\mathrm{NMA}}$ and a translation vector $\mathbf{d}_i^{\mathrm{NMA}}$. The three rotational DOFs $u_i^{rx}, u_i^{ry}, u_i^{rz}$ are written in the axis-angle representation with rotation angle

$$\theta_i = \sqrt{(u_i^{rx})^2 + \left(u_i^{ry}\right)^2 + (u_i^{rz})^2} \qquad [3]$$

and rotation axis

$$\mathbf{a}_i = \left(a_i^x, a_i^y, a_i^z\right)^{\mathrm{T}} = \left(u_i^{rx}, u_i^{ry}, u_i^{rz}\right)^{\mathrm{T}} / \theta_i \qquad [4]$$

In Eq. [2], the rotation matrix is equivalent to the axis-angle representation,

$$\mathbf{R}_i^{\mathrm{NMA}} = \begin{pmatrix} \cos\theta_i + (1-\cos\theta_i)(a_i^x)^2 & (1-\cos\theta_i)a_i^x a_i^y - a_i^z \sin\theta_i & (1-\cos\theta_i)a_i^x a_i^z + a_i^y \sin\theta_i \\ (1-\cos\theta_i)a_i^x a_i^y + a_i^z \sin\theta_i & \cos\theta_i + (1-\cos\theta_i)\left(a_i^y\right)^2 & (1-\cos\theta_i)a_i^y a_i^z - a_i^x \sin\theta_i \\ (1-\cos\theta_i)a_i^x a_i^z - a_i^y \sin\theta_i & (1-\cos\theta_i)a_i^y a_i^z + a_i^x \sin\theta_i & \cos\theta_i + (1-\cos\theta_i)(a_i^z)^2 \end{pmatrix} \qquad [5]$$

and the translation vector is given by

$$\mathbf{d}_i^{\mathrm{NMA}} = \left(u_i^{tx}, u_i^{ty}, u_i^{tz}\right)^{\mathrm{T}}. \qquad [6]$$

As an illustrative example, we modeled a flexible B-form DNA duplex with 48 basepairs designed by Wozniak et al. (4). Two dyes, which are AF488 as the donor and Cy5 as the acceptor, are tethered to the C7 atoms in thymines via flexible linkers. The two attachment atoms are separated by 27 basepairs. NMA is applied to generate a set of 1,000 structures, which provide the physical fluctuations of Euclidean distances between the attachment atoms as shown in Figure S3. For the 1,000 structures, the standard deviation of the Euclidean distance between the two attachment atoms is 0.28 nm.

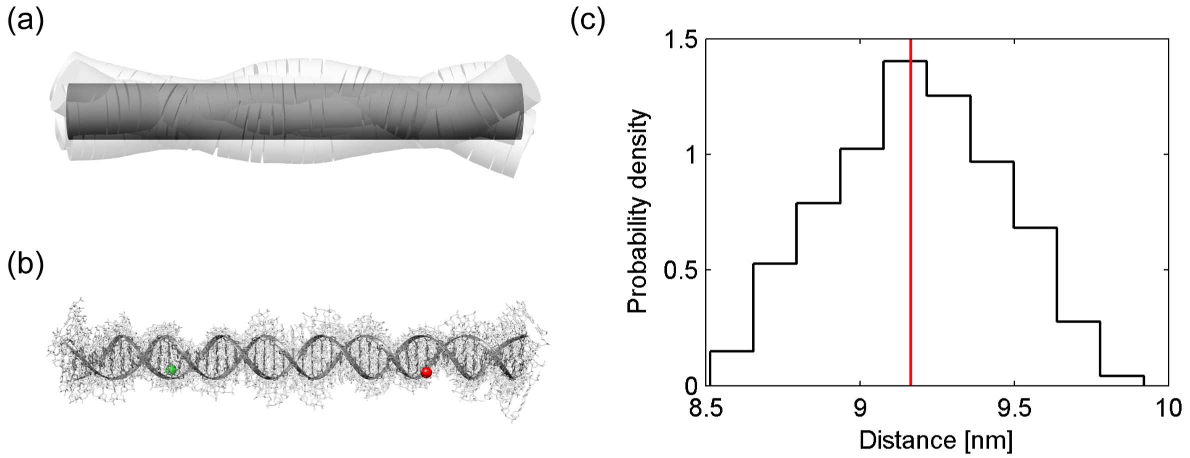

Figure S3. (a) The finite element model of a B-form DNA duplex with the mechanical ground-state structure (dark gray) superposed on the flexible structures (light gray). (b) The corresponding atomic model with the same coloring as in (a). The attachment atoms of AF488 and Cy5 are colored in green and red, respectively. (c) Distribution of the Euclidean distance between attachment atoms in the atomic model of the flexible structures. The red vertical line shows the same distance in the atomic model of the mechanical ground-state structure.

SUPPLEMENTARY NOTE 3: Dye modeling

To model FRET, we calculate the distance $\boldsymbol{r}_{ij}$ and orientation factor κ_{ij} between each pair of dyes. The transition dipole moment of each dye is denoted by its unit vector $\hat{\mathbf{d}}_i$ and center $\mathbf{r}_i$, which are determined by the dye atomic structure and environment. In the present work, base-substituted, intercalating, and tethered dyes are modeled.

Intercalating and base-substituted dye models

The unit transition dipole vector $\hat{\mathbf{d}}_i$ and dipole center $\mathbf{r}_i$ are assumed to be known from quantum-based electronic structure calculation. In general, two orientational degrees of freedom, θ and ϕ, of the dipole vector specify its conformation relative to the local basepair, with axes perpendicular and parallel to the reference basepair plane, respectively, using the same convention as the software FRET-matrix (9). The degrees of freedom θ and ϕ are defined in Figure 2a of Preus et al. (9). FRET-matrix additionally specifies the conformational distribution of states that account for dye flexibility, modeled as the independent distributions $p(\theta)$ and $p(\phi)$. In the present work we ignore local dye conformational flexibility in assuming that the DNA conformational flexibility dominates the dipole orientational distribution of states for intercalating and base-substituted dyes. When conformational flexibility is included in the calculation of FRET between two dyes intercalated in DNA, the instantaneous orientations of the dye dipole vectors are fixed relative to the atomic structure computed according to Supplementary Note 2. Because FRET is assumed to be faster than the time-scale of these large-scale conformational fluctuations of DNA itself, static averaging may be performed. If fast time-scale, local dye conformational fluctuations are also accounted for, an additional layer of isotropic, static or dynamic averaging must be performed, depending on the time-scale of the local dye conformational fluctuations compared to the FRET rate.

Tethered dye model

Tethered dyes are covalently linked to attachment atoms in DNA via flexible linkers. Figure 3 in the main text shows a number of chemical structures for the tethered dyes and linkers studied in the present work. These dyes are typically employed in single-molecule spectroscopy to resolve the structure and conformational states of molecules. Flexible linkers are typically employed in order to yield an isotropic rotational distribution of states so that $\langle\kappa^2\rangle = 2/3$ may be assumed. Here, we assume that these dyes are

randomly oriented with respect to DNA and that they sample the full, sterically accessible volume (AV) in the neighborhood of their attachment point (13). The AV algorithm relies on the following modeling parameters for each dye:

1. The atomic structure of the DNA nanostructure and the van der Waals radius of each atom.
2. The linker attachment atom on DNA.
3. The dye radius, linker length, and linker width, which are geometric parameters that describe the size and the accessible volume of the dye (Table S2).

The AV algorithm is then used to generate a 3D grid about the linker attachment atom on DNA. Grid points clash with DNA and are excluded from the AV if either of the two conditions below is satisfied:

1. The Euclidean distance between the grid point and any DNA atom is less than the sum of the radius of the dye and the van der Waals radius of the DNA atom.
2. The Euclidean distance between this grid point and the attachment point is greater than the width of the linker, and the Euclidean distance between this grid point and any DNA atom is less than the sum of the van der Waals radius of this DNA atom and the linker half-width.

Then the AV algorithm attempts to route the flexible linker from the attachment atom to each of the grid points that do not clash with DNA (13). A route for the flexible linker consists of a series of grid points with no steric clash with DNA. The AV is defined as the set of grid points to which a route for the flexible linker exists.

The AV provides the ensemble of positions where the dye can be positioned at a given time. The orientation of the dye is assumed random. For FRET calculations, this AV is sampled, and the FRET efficiency is averaged with a procedure that depends on the time-scale at which the AV is explored by the dye, relative to the FRET rate. This method can be a poor approximation in the case of dyes that interact chemically with DNA. However, this is still the best approximation available besides performing MD simulations, which will be the subject of future work.

Table S2. Geometric parameters of covalently tethered dyes with flexible linkers. The parameters without references were estimated with the software Avogadro, Version 1.1.0 (14).

DNA nanostructure	Dye	Dye radius [Å]	Linker length [Å]	Linker width [Å]
DNA-FRET-pair (4)	AF488	3.5 (13)	20.0 (13)	4.5 (13)
	Cy5	3.5 (13)	23.0 (13)	4.5 (13)
DNA Wire (5)	PB	2.5	16.0	4.5 (13)
	Cy3	3.5	18.0	4.5 (13)
Seven-helix dsDNA bundle (6)	Cy3	3.5	9.0	4.5 (13)
	AF647	3.5	19.0	4.5 (13)

SUPPLEMENTARY NOTE 4: FRET orientation factor models

The orientation factor describing the FRET between two interacting dyes adopts a range of values depending on the dye configurations sampled. Figure S4 explores some common cases. (a) to (c) show the orientation factor for coplanar, fixed, parallel dyes. When the transition dipole moments are collinear and placed side-by-side, the orientation factor is 1. The maximal value of 4 is reached when both transition dipole moments are collinear in the head-to-head configuration (b). However this situation can also lead to a value of 0 if the angle Ω satisfies the condition $sin(\Omega) = 1/\sqrt{3}$. The orientation factor is thus shown to strongly vary not only from the relative orientation of the dipoles, but also from their relative position. (d) to (e) show similar results for co-planar dyes that are perpendicular in a collinear configuration. (g) and (h) involve freely rotating, un-restricted dyes. When both dyes are freely rotating, the orientation factor is a fixed value, independent of the position of the dyes. However, when a fixed dye interacts with a freely rotating dye, dependence on the position is recovered. More general cases involving freely rotating, un-correlated, dyes restricted to conical surfaces and volumes can be found in the literature (15-17). Position and orientation of dyes in a FRET pair thus strongly affects the FRET rate, which may in turn also strongly affect the FRET efficiency.

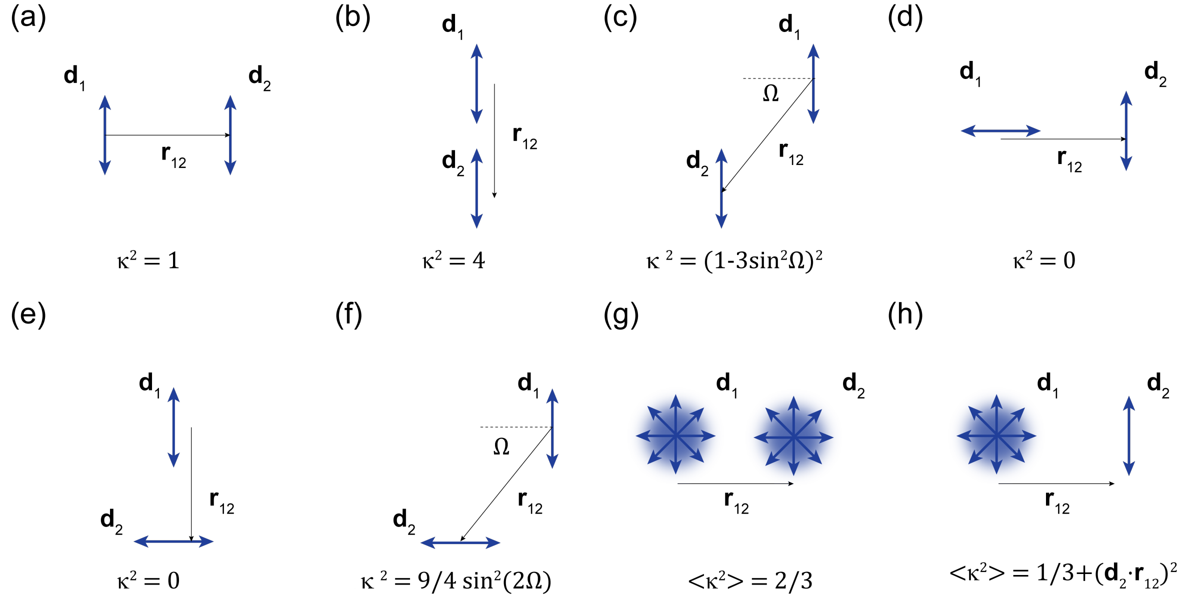

Figure S4. (a)-(f) Orientation factor $\langle \kappa^2 \rangle$ for fixed co-planar dyes. (g) Orientation factor between two freely rotating, uncorrelated, unrestricted dyes. (h) Orientation factor between a freely rotating, uncorrelated, unrestricted dye and a fixed dye (17).

SUPPLEMENTARY NOTE 5: Dye photophysical properties and DAS calculation

Dye photophysical properties relevant to our modeling consist of the quantum yield, fluorescence lifetime, and molar extinction. Quantum yield and lifetime for Cy3/7HB, AF647 and Pyrene (Py) are from (6). Quantum yield and lifetime for Pacific blue (PB) and Cy3 are from (5). Lifetime and quantum yield for YO-PRO-1 are from (18). Lifetime, quantum yield and molar extinction coefficients for ATTO dyes are from the ATTO-TEC website (www.atto-tec.com). Molar extinction for Py is from unpublished experimental data. Other molar extinction coefficients and quantum yield and lifetime for AF750 are from the spectra database hosted at the University of Arizona (www.spectra.arizona.edu).

Table S3. Photophysical properties of individual dyes

DNA nanostructure	Dye	Quantum yield	Fluorescence lifetime [ns]	Molar extinction [$M^{-1}cm^{-1}$ @ nm]
DNA Wire	PB	0.75	2.6	46000@416
	YO-PRO-1	0.48[a]	3.37[b]	52000@491
	Cy3	0.16	0.3	150000@550
Seven-helix dsDNA bundle	Cy3	0.23[c]	1.1[c]	150000@550
	AF647	0.33	0.7[c]	239000@649
	Py	0.1[c]	1.2[c]	37573@380
DNA FRET switch	ATTO488	0.82	3.1	90000@501
	ATTO565	0.92	3.4	120000@563
	ATTO647N	0.65	3.2	150000@644
	AF750	0.12	0.7	240000@749

[a] This value is for $y = 0$. (y being number of YO/basepair). For other y, $QY = QY_0\left(0.73e^{-y/0.26} + 0.27\right)$.
[b] This value is for $y = 0$. For other y, $\tau = \tau_0\left(0.35e^{-y/0.2} + 0.65\right)$.
[c] Those values have been measured for dyes attached to DNA.

Förster radii between pairs of dyes for the 7HB are evaluated from their emission and absorption spectrum and quantum yield. The refractive index of the environment is assumed to be 1.4. The Förster radius for the AF488/Cy5 pair on the dsDNA wire is taken from (4). The Förster radius for the PB/YO-PRO-1 pair and the YO-PRO-1/Cy3 pair on the dsDNA is from (5). The YO-PRO-1 homo-FRET radius is from (18). Radii for Py/Cy3, Cy3/AF647, Py/AF647 and dyes part of the FRET switch have been calculated from their photophysical properties.

Table S4. Förster radius between dye pairs

DNA nanostructure	Donor	Acceptor	Förster radius [Å]
Seven-helix dsDNA bundle	Py	Cy3	37
	Cy3	AF647	55
	Py	AF647	33
	Cy3	Py	26
	AF647	Py	29
	Py	Py	24
	Cy3	Cy3	47
DNA-FRET-pair	AF488	Cy5	52
DNA wire	PB	YO-PRO-1	52
	PB	Cy3	51
	YO-PRO-1	YO-PRO-1	43[a]
	YO-PRO-1	Cy3	59[a]
DNA FRET switch	ATTO488	ATTO565	62
	ATTO488	ATTO647N	51
	ATTO488	AF750	45
	ATTO565	ATTO488	26
	ATTO565	ATTO565	58
	ATTO565	ATTO647N	67
	ATTO565	AF750	63
	ATTO647N	ATTO488	19
	ATTO647N	ATTO565	10
	ATTO647N	AF750	79

[a] This value is for $y = 0$. For other y, variation in the quantum yield and lifetime presented in Table S3 must be considered.

The Decay Associated Spectrum (DAS) is used to report the wavelength dependent fluorescence time-resolved signal as a superposition of decays associated to distinct time constants.

$$\varphi(\lambda, t) = \sum_{i} DAS_i(\lambda) e^{-\alpha_i t} \quad [7]$$

The DAS is calculated from the rate matrix $\mathbf{K}$ that contains all pairwise FRET rates, including their self-decay. The decay of each dye following initial excitation is then given by,

$$\frac{d\boldsymbol{\rho}}{dt} = \mathbf{K}\boldsymbol{\rho}. \quad [8]$$

This equation is solved by diagonalizing the $\mathbf{K}$ matrix using the eig function in MATLAB. This process yields the diagonal matrix $\mathbf{D}$ that contains the eigenvalues $-\alpha_i$, and the eigenvectors $\boldsymbol{\beta}_i$. The solution of the differential equation may then be written,

$$\boldsymbol{\rho} = \sum_i c_i \boldsymbol{\beta}_i e^{-\alpha_i t}. \quad [9]$$

The coefficients c_i are calculated from the initial condition and the transformation matrix that diagonalizes $\mathbf{K}$. Knowing the emission spectrum, quantum yield and fluorescence lifetime of each individual dye, the spectrum associated with each particular value α_i can be calculated from the solution of Eq. [9], yielding the DAS. Note that Eq. [9] is valid only if the eigenvalues $-\alpha_i$ are distinct. Situations with repeated eigenvalues are outside the scope of this work.

SUPPLEMENTARY NOTE 6: FRET in flexible DNA wires

Conformational fluctuations of DNA affect the relative orientations and positions of the intercalated dyes in the DNA wire. However, apart from some rare cases, the strongest coupling of a particular dye generally occurs with its nearest neighbors, despite this fluctuation. Significant variation in the FRET rate between those dyes occurs. However, their average FRET rate remains similar to the rigid wire case. Since the FRET rate remains much higher than the fluorescence rate, the overall transfer efficiency is only weakly affected.

(a) 50-mer DNA wire

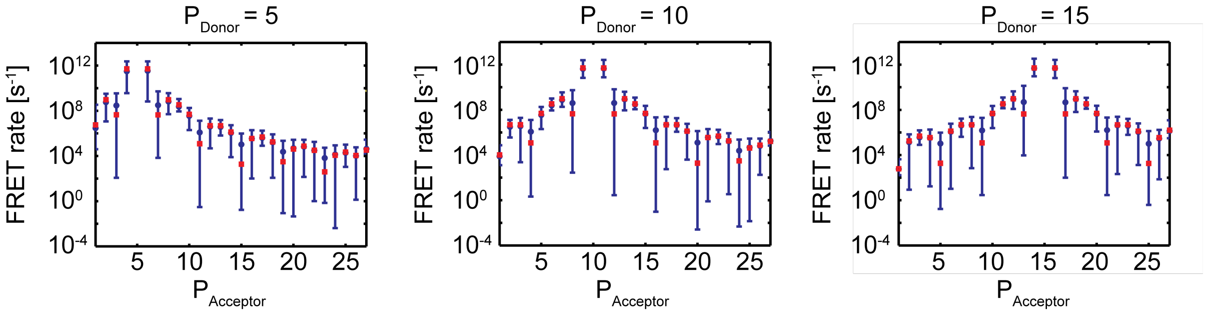

(b) 20-mer DNA wire

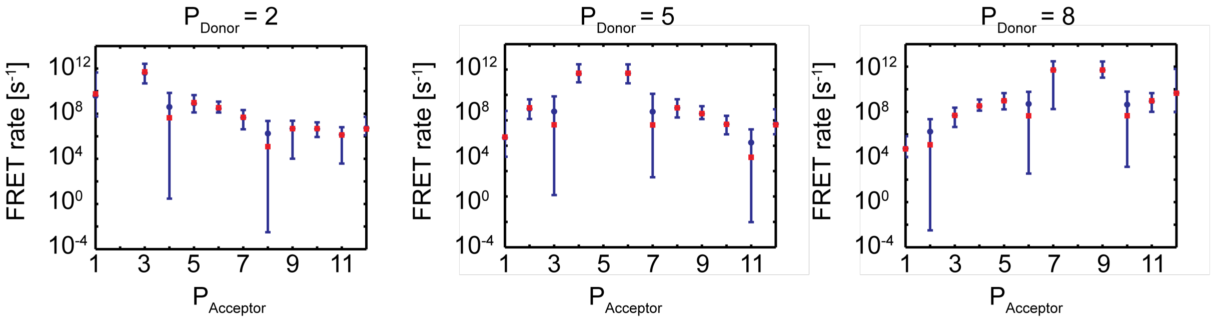

Figure S5. (a) Pairwise FRET rates between neighboring dyes in the 50-mer wire at a concentration of 0.5 YO/basepair. This wire includes a PB dye at position 1, YO dyes at positions 2-26 and a Cy3 dye at position 27. Blue dots represent the average FRET rates between the dye situated at P_{Donor} and the one situated at $P_{Acceptor}$ for a DNA wire with conformational flexibility. The blue bars shows the maximum and minimum FRET rates reached in the different conformations. Red squares show the FRET rates in the rigid wire. (b) Similar to (a), but for a 20-mer DNA wire. This wire includes a PB dye at position 1, YO dyes at positions 2-11 and a Cy3 dye at position 12.

SUPPLEMENTARY NOTE 7: Static averaging for the seven-helix dsDNA bundle light-harvesting antenna

FRET lifetimes between dyes in the seven-helix dsDNA bundle light-harvesting antenna are in the range of 0.8 ps to 12.5 ns and 0.5 ps to 33 ns, for the 1:1:1 and 6:6:1 antenna, respectively. These lifetimes can get significantly shorter than typical dye rotational lifetime $\tau_R \sim$ 0.1−1 nsec, and static averaging instead of isotropic averaging could arguably be more justified for some dye pairs. However, considering static averaging introduces only minor modifications to the steady-state absorption and emission spectra (Figure S6) and slight modification of the antenna effect (Table S5) compared to the results reported in the main text that consider isotropic averaging.

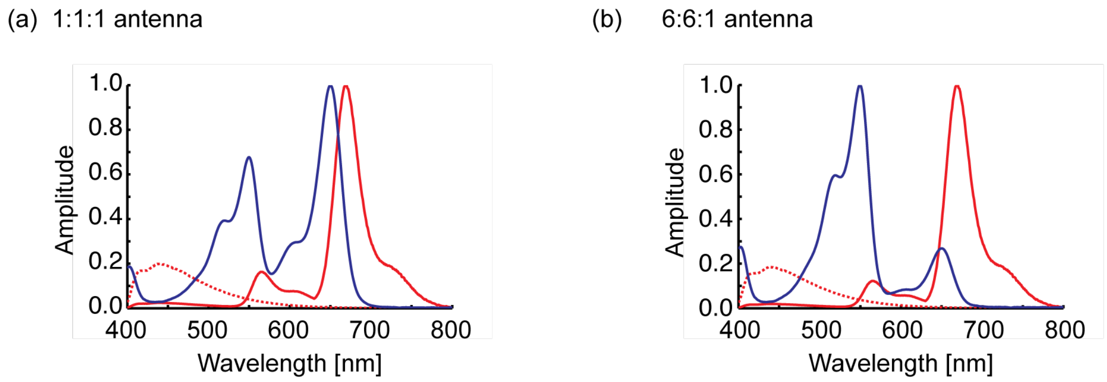

Figure S6. Steady-state absorption (blue line) and emission (red line) spectra for the 1:1:1 (a) and 6:6:1 (b) seven-helix bundle light-harvesting antenna. For the emission spectrum, initial excitation of Py is assumed. Dot red line is the emission spectrum of Py in the absence of other dyes. Static averaging is assumed.

Table S5. Antenna effect of the seven-helix bundle antenna with static averaging

**Configuration (Py:Cy3:AF)**	**Antenna Effect**
6:6:1	2.62
1:1:1	0.36

SUPPLEMENTARY NOTE 8: Potential sources of discrepancy between theoretical and experimental seven-helix dsDNA bundle antenna light-harvesting results

Anisotropy measurements presented by Dutta et al. (6) suggest that the isotropic approximation for dye orientations assumed in our modeling may not be justified, particularly for Cy3, which is known to have limited mobility when attached to the sugar-phosphate backbone of DNA (19,20). The orientation factor $\langle\kappa^2\rangle$ may in this case change significantly, varying from 0−4 instead of the 2/3 assumed (see Supplementary Note 4). However, Cy3-AF distances are much smaller (3.1 nm and 1.8−4.3 nm for the 1:1:1 and 6:6:1 antenna designs, respectively) than the Förster radius of the dye pair (5.5 nm for $\langle\kappa^2\rangle = 2/3$), so that small variations in $\langle\kappa^2\rangle$ from its isotropic value do not significantly affect the Cy3-AF transfer efficiency. Specifically, in order to account for the experimentally observed antenna effect published by Dutta et al. for the 1:1:1 antenna (6), $\langle\kappa^2\rangle$ for the Cy3-AF and Py-AF pairs must be reduced to approximately 0.01 (Figure S7). Given the small inter-dye separation, this value imposes a rather restricted mobility and orientation of the interacting transition dipoles of Py, Cy3 and AF. A similar situation occurs for the 6:6:1 antenna, where $\langle\kappa^2\rangle$ would need to be further reduced to approximately 0.001 for all Cy3-AF and Py-AF pairs to account for the experimentally observed antenna effect (Figure

S7). If we allow modifying $\langle\kappa^2\rangle$ also for the Py-Cy3 pairs, then $\langle\kappa^2\rangle$ must be reduced to ~0.01 for both the 6:6:1 and 1:1:1 antenna to account for the experimental antenna effect. However, the calculated quenching efficiency of Py then drops from ~95% to ~60%, which compares badly with the experimental data (6). Consequently, the theoretical calculations performed here suggest that alternative sources of experimental variability must be invoked to account for the relatively low transfer efficiency between Cy3 and AF observed experimentally. Several possibilities include, but are not limited to, the breakdown at short distances of the point dipole approximation employed in the FRET model (21), changes in the photophysical properties of the tethered dyes (22), or defects in the experimentally synthesized antennas that are not represented by the model, such as missing dyes or defective DNA nanostructures. Single-molecule FRET measurements in addition to a more detailed theoretical treatment may be of interest to explore these questions in more detail in separate work.

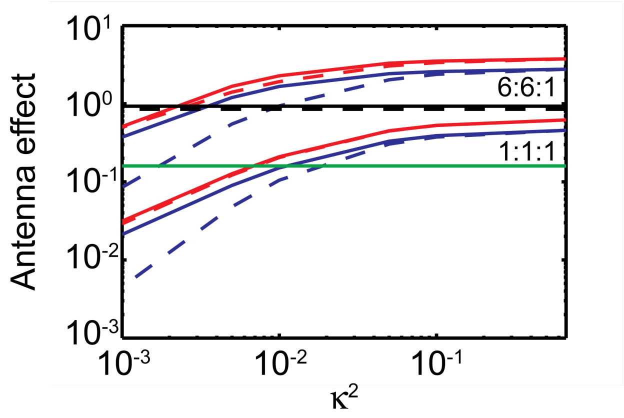

Figure S7. Variation of the antenna effect as a function of the orientation factor for the 6:6:1 and 1:1:1 seven-helix dsDNA bundle antenna. Red line is the calculated antenna effect upon excitation of Py, denoted AE1 in (6) and the blue line is the antenna effect upon excitation of Cy3, denoted AE2 in (6). Solid lines show the variation when only the orientation factor of Py-AF and Cy3-AF pairs are affected, whereas dashed lines include also variation of the orientation factors of Py-Cy3 pairs. Solid and dashed black lines are the experimental result for AE1 and AE2, respectively, for the 6:6:1 seven-helix dsDNA bundle antenna. Solid and dashed green lines are the experimental result for AE1 and AE2, respectively, for the 1:1:1 seven-helix dsDNA bundle antenna.

SUPPLEMENTARY NOTE 9: Benchmark for the FRET calculation

FRET model have been used to determine FRET transfer over a DNA wire including an increasing number of dyes to benchmark its performance. Both the steady-state and time-dependent solution have been computed. FRET transfer in wires composed of more than 10,000 dyes can be computed in under an hour on an Intel Xeon X5690 workstation.

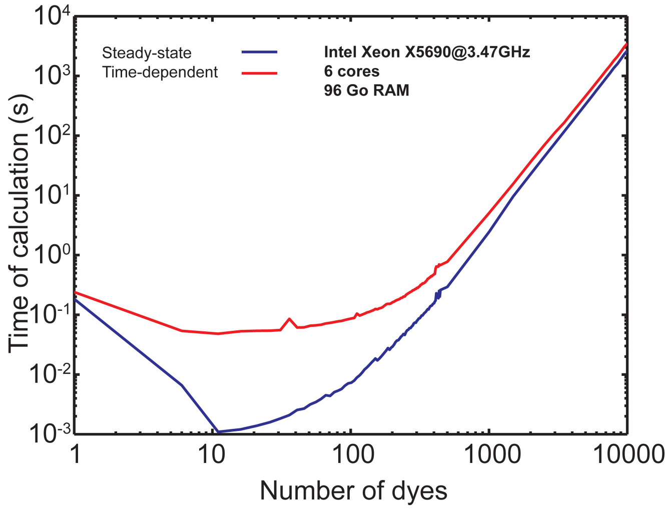

Figure S8. Calculation time for the FRET as a function of the number of dyes involved on a Xeon workstation.

REFERENCES

1. Kim, D.N., Kilchherr, F., Dietz, H. and Bathe, M. (2012) Quantitative prediction of 3D solution shape and flexibility of nucleic acid nanostructures. *Nucleic Acids Research*, **40**, 2862-2868.
2. Castro, C.E., Kilchherr, F., Kim, D.N., Shiao, E.L., Wauer, T., Wortmann, P., Bathe, M. and Dietz, H. (2011) A primer to scaffolded DNA origami. *Nature Methods*, **8**, 221-229.
3. Douglas, S.M., Marblestone, A.H., Teerapittayanon, S., Vazquez, A., Church, G.M. and Shih, W.M. (2009) Rapid prototyping of 3D DNA-origami shapes with caDNAno. *Nucleic Acids Research*, **37**, 5001-5006.
4. Wozniak, A.K., Schroder, G.F., Grubmuller, H., Seidel, C.A.M. and Oesterhelt, F. (2008) Single-molecule FRET measures bends and kinks in DNA. *Proceedings of the National Academy of Sciences of the United States of America*, **105**, 18337-18342.
5. Hannestad, J.K., Sandin, P. and Albinsson, B. (2008) Self-assembled DNA photonic wire for long-range energy transfer. *Journal of the American Chemical Society*, **130**, 15889-15895.
6. Dutta, P.K., Varghese, R., Nangreave, J., Lin, S., Yan, H. and Liu, Y. (2011) DNA-directed artificial light-harvesting antenna. *Journal of the American Chemical Society*, **133**, 11985-11993.
7. Lu, X.J. and Olson, W.K. (2003) 3DNA: a software package for the analysis, rebuilding and visualization of three-dimensional nucleic acid structures. *Nucleic Acids Research*, **31**, 5108-5121.
8. Olson, W.K., Bansal, M., Burley, S.K., Dickerson, R.E., Gerstein, M., Harvey, S.C., Heinemann, U., Lu, X.J., Neidle, S., Shakked, Z. *et al.* (2001) A standard reference frame for the description of nucleic acid base-pair geometry. *Journal of Molecular Biology*, **313**, 229-237.
9. Preus, S., Kilsa, K., Miannay, F.A., Albinsson, B. and Wilhelmsson, L.M. (2013) FRETmatrix: a general methodology for the simulation and analysis of FRET in nucleic acids. *Nucleic Acids Research*, **41**, e18.

10. Günther, K., Mertig, M. and Seidel, R. (2010) Mechanical and structural properties of YOYO-1 complexed DNA. *Nucleic Acids Research*, **38**, 6526-6532.
11. Carlsson, C., Larsson, A., Jonsson, M., Albinsson, B. and Norden, B. (1994) Optical and photophysical properties of the oxazole yellow DNA probes YO and YOYO. *The Journal of Physical Chemistry*, **98**, 10313-10321.
12. Spielmann, H.P., Wemmer, D.E. and Jacobsen, J.P. (1995) Solution structure of a DNA complex with the fluorescent bis-intercalator TOTO determined by NMR-spectroscopy. *Biochemistry*, **34**, 8542-8553.
13. Kalinin, S., Peulen, T., Sindbert, S., Rothwell, P.J., Berger, S., Restle, T., Goody, R.S., Gohlke, H. and Seidel, C.A.M. (2012) A toolkit and benchmark study for FRET-restrained high-precision structural modeling. *Nature Methods*, **9**, 1218-1225.
14. Hanwell, M.D., Curtis, D.E., Lonie, D.C., Vandermeersch, T., Zurek, E. and Hutchison, G.R. (2012) Avogadro: an advanced semantic chemical editor, visualization, and analysis platform. *Journal of Cheminformatics*, **4**, 1-17.
15. Corry, B.B., Jayatilaka, D.D., Martinac, B. and Rigby, P. (2006) Determination of the orientational distribution and orientation factor for transfer between membrane-bound fluorophores using a confocal microscope. *Biophysical Journal*, **91**, 1032-1045.
16. Dale, R.E., Eisinger, J. and Blumberg, W.E. (1979) The orientational freedom of molecular probes. *Biophysical Journal*, **26**, 161-194.
17. Dale, R.E. and Eisinger, J. (1974) Intramolecular distances determined by energy transfer. Dependence on orientational freedom of donor and acceptor. *Biopolymers*, **13**, 1573-1605.
18. Woller, J.G., Hannestad, J.K. and Albinsson, B. (2013) Self-assembled nanoscale DNA-porphyrin complex for artificial light harvesting. *Journal of the American Chemical Society*, **135**, 2759-2768.
19. Ranjit, S., Gurunathan, K. and Levitus, M. (2009) Photophysics of backbone fluorescent DNA modifications: reducing uncertainties in FRET. *Journal of Physical Chemistry B*, **113**, 7861-7866.
20. Spiriti, J., Binder, J.K., Levitus, M. and van der Vaart, A. (2011) Cy3-DNA stacking interactions strongly depend on the identity of the terminal basepair. *Biophysical Journal*, **100**, 1049-1057.
21. Khan, Y.R., Dykstra, T.E. and Scholes, G.D. (2008) Exploring the Förster limit in a small FRET pair. *Chemical Physics Letters*, **461**, 305-309.
22. Levitus, M. and Ranjit, S. (2011) Cyanine dyes in biophysical research: the photophysics of polymethine fluorescent dyes in biomolecular environments. *Quarterly Reviews of Biophysics*, **44**, 123-151.
