# Supplementary material for: Structure-based model for light-harvesting properties of nucleic acid nanostructures
Source: Nucleic Acids Res. 2013 Dec 5;42(4):2159–70. doi: 10.1093/nar/gkt1269 (PMC3936709; doi:10.1093/nar/gkt1269)
Supplement: Supplementary Data [file supp_gkt1269_suppl_data.zip › nar-02976-f-2013-File010.zip!/Cando-FRET/Manual_FRETopt.pdf]

Documentation of the software CanDo-FRET for structure-based FRET calculation

Keyao Pan, Etienne Boulais, Lun Yang, and Mark Bathe
Laboratory for Computational Biology & Biophysics
Department of Biological Engineering
Massachusetts Institute of Technology
Cambridge, MA 02139, USA

Syntax

[A,rhoj,T,rho,eff,avg_TE] = FRETopt(D,n,R,t_end,R0,tau,mol_ext,kind,ss,random,pos_don,pos_acc,par)

Input parameters

Parameter	Dimension	Description
n	Scalar	Scalar defining the number of dyes present in the system.
R	n×3	Matrix containing the positions of the center of the transition dipole of each dye.
D	n×3	Matrix containing the unit vector orientation of the transition dipole of each dye.
T_end	Scalar	Scalar defining the total time of the simulation.
R0	nt×nt	Matrix containing the Foerster radius for each distinct pair of dyes. nt is the total number of dye types present in the system. R0(i,j) defines the Foerster radius for a donor of type i and an acceptor of type j.
tau	1×nt	Matrix defining the fluorescence lifetime of each type of dye.
mol_ext	1×nt	Matrix defining the molar extinction of each type of dye at the irradiation wavelength.
kind	1×n	Matrix that defines the type of each dye.
ss	Scalar	Scalar flag defining whether the system of equations is solved in steady-state (ss=1) or not (time-dependent).
random	1×…	Matrix specifying which dyes are assumed to have isotropic, random orientation.
pos_don	1×…	Matrix specifying which dye is assumed to be an initial donor dye.
pos_acc	1×…	Matrix specifying which dye is assumed to be a final acceptor dye.
par	Scalar	Scalar flag specifying whether parallelization (par=1) of the matrix assembly is used.

Output parameters

Parameter	Dimension	Description
avg_TE	Scalar	Scalar storing the transmission efficiency.
eff	Scalar	Scalar storing the quenching efficiency of donors.
rho	n×1 (ss=1)<br>n×ts (ss=0)	Matrix that stores the exciton population corresponding to each dye.
T	n×ts	Matrix that stores the time steps used by the solver.
rhoj	nt×1	Matrix that stores the initial condition used by the solver.
A	n×n	Matrix that stores the rate matrix used by the solver (corresponding to matrix K in the main manuscript)
